# Improved automatic steam distillation combined with oscillation-type densimetry for determining alcoholic strength in spirits and liqueurs

**DOI:** 10.1186/s40064-015-1574-6

**Published:** 2015-12-18

**Authors:** Dirk W. Lachenmeier, Leander Plato, Manuela Suessmann, Matthew Di Carmine, Bjoern Krueger, Armin Kukuck, Markus Kranz

**Affiliations:** Chemisches und Veterinäruntersuchungsamt (CVUA) Karlsruhe, Weissenburger Strasse 3, 76187 Karlsruhe, Germany; Lab Synergy, 374 Pulaski Highway, Goshen, NY 10924 USA; C. Gerhardt GmbH & Co. KG, Cäsariusstraße 97, 53639 Königswinter, Germany

**Keywords:** Ethanol, Alcoholic strength, Steam distillation, Oscillation-type densimetry, Experimental design, Optimisation, Validation

## Abstract

The determination of the alcoholic strength in spirits and liqueurs is required to control the labelling of alcoholic beverages. The reference methodology prescribes a distillation step followed by densimetric measurement. The classic distillation using a Vigreux rectifying column and a West condenser is time consuming and error-prone, especially for liqueurs that may have problems with entrainment and charring. For this reason, this methodology suggests the use of an automated steam distillation device as alternative. The novel instrument comprises an increased steam power, a redesigned geometry of the condenser and a larger cooling coil with controllable flow, compared to previously available devices. Method optimization applying D-optimal and central composite designs showed significant influence of sample volume, distillation time and coolant flow, while other investigated parameters such as steam power, receiver volume, or the use of pipettes or flasks for sample measurement did not significantly influence the results. The method validation was conducted using the following settings: steam power 70 %, sample volume 25 mL transferred using pipettes, receiver volume 50 mL, coolant flow 7 L/min, and distillation time as long as possible just below the calibration mark. For four different liqueurs covering the typical range of these products between 15 and 35 % vol, the method showed an adequate precision, with relative standard deviations below 0.4 % (intraday) and below 0.6 % (interday). The absolute standard deviations were between 0.06 % vol and 0.08 % vol (intraday) and between 0.07 % vol and 0.10 % vol (interday). The improved automatic steam distillation devices offer an excellent alternative for sample cleanup of volatiles from complex matrices. A major advantage are the low costs for consumables per analysis (only distilled water is needed). For alcoholic strength determination, the method has become more rugged than before, and there are only few influences that would lead to incomplete distillation. Our validation parameters have shown that the performance of the method corresponds to the data presented for the reference method and we believe that automated steam distillation, can be used for the purpose of labelling control of alcoholic beverages.

## Background

In the regulatory control of alcoholic beverages, the determination of the alcoholic strength is considered as pivotal parameter (Lachenmeier et al. 2010). Sample preparation by distillation followed by some form of density measurement (i.e. pycnometry, electronic densimetry and densimetry using hydrostatic balance) is still considered the gold standard and the reference methodology for determination of alcoholic strength (European Commission 2000; OIV 2009), even when more rapid and direct methods such as infrared spectroscopy are gaining more and more distribution in routine testing laboratories (López Mahía et al. 1992; Gallignani et al. 1993, 1994a, b, c; Maudoux et al. 1998; Lachenmeier et al. 2010; Lachenmeier 2007).

For density measurement, electronic densimetry based on electromagnetically-induced oscillation of a U-shaped glass tube, is superior in performance and accuracy compared to pycnometry, hydrostatic balance or hydrometry (Strunk et al. 1979; Mark and Vaughn 1980; Kovár 1981; Brereton et al. 2003).

The distillation step before the densimetric measurement is required to remove sugars and other solutes that would otherwise lead to false results, as the tables for converting density to alcoholic strength are based on pure water-alcohol mixtures (European Commission 2000; OIV 2009). Only for some very clean spirits such as vodka a direct densimetric measurement may be feasible.

The EU reference method states some specifications for the distillation, which include that the apparatus must be leak-tight, the regularisation of the distillation rate must be possible, a rapid and complete condensation of the alcohol vapours must occur, and the first distillation fractions must be collected in an aqueous medium. As example, the EU reference method provides a classical distillation apparatus consisting of a 20-cm Vigreux rectifying column, a 10-cm straight-rimmed West condenser (a variant of a Liebig condenser), and a 40-cm cooling coil (European Commission 2000). However, other suitable distillation devices may be applied, e.g. such as specified by IUPAC (1968). The only method performance requirement for the distillation apparatus is that the distillation of 200 mL of a water-alcohol solution with known concentration close to 50 % vol must not cause a loss of alcohol of more than 0.1 % vol (European Commission 2000). The classical distillation is comparably time-consuming, difficult to automate and problematic for some samples (such as liqueurs) that may have problems with entrainment and charring. For these reasons, our group has introduced the use of automatic steam distillation for the purpose in 2003 (Lachenmeier et al. 2003) [English summary version published in 2004 (Lachenmeier 2004)]. Using optimised settings of the steam-distillation device, the method had a wide application range for alcoholic beverages between 2 and 80 % vol with identical results to classical distillation. In follow-up studies, we have shown the applicability for complex matrices such as egg liqueurs (Lachenmeier et al. 2005), alcohol powders (Bauer-Christoph and Lachenmeier 2005) or cherry-spirit containing cakes (Lachenmeier et al. 2007).

Following the initial experiments with steam distillation, instruments with increased steam power became available showing a larger optimal range independent of specific settings such as distillation time, alcoholic strength of sample, sample volume or receiver volume (Lachenmeier et al. 2006). In this paper, we introduce the use of a completely new steam distillation device. The device not only comprises an increased steam power but also a redesigned geometry of the condenser, as well as a larger cooling coil with controllable flow. Based on a method development and validation study, this paper describes the optimized procedure for alcoholic strength determination using steam distillation.

## Methods

### Instrumentation

**Fig. 1:**
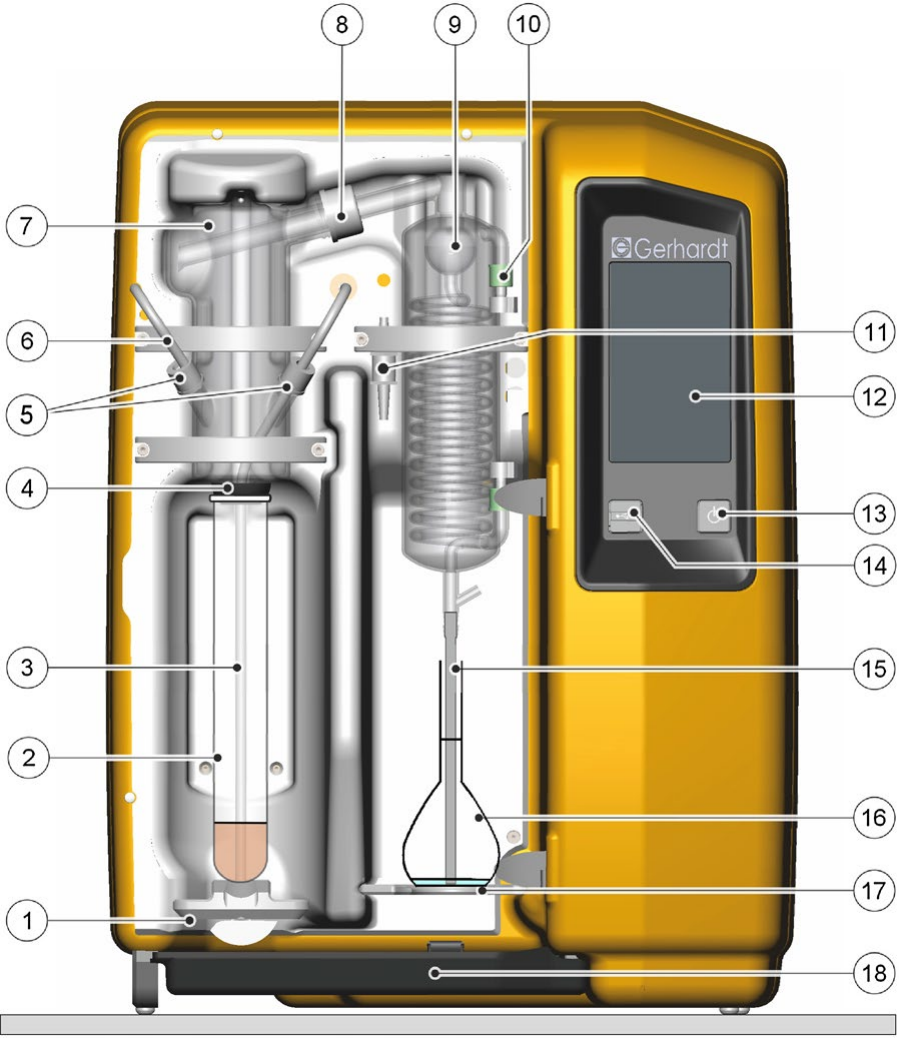
Automated steam distillation device with setup for the determination of alcoholic strength in spirits. (*1*) Quick clamping device with clamping block, (*2*) Kjeldatherm digestion tube with sample, (*3*) PTFE steam inlet tubing, (*4*) Viton connection stopper, (*5*) screw caps, (*6*) NaOH inlet (closed with blind cap for alcohol distillation), (*7*) glass distribution head, (*8*) screw cap, (*9*) glass distillation condenser cooled at 10 °C, (*10*) screw cap, (*11*) ventilation valve, (*12*) control panel, (*13*) standby switch, (*14*) USB interface, (*15*) silicone outlet tubing for distillate discharge, (*16*) graduated flask as receiver (filled with small volume of water, in which the outlet tubing must be submerged), (*17*) receiver table, (*18*) drip tray

The automated steam distillations were facilitated with the Gerhardt Vapodest 200 (new device, Fig. 1) in comparison to the Gerhardt Vapodest 30 [old device used in previous studies (Lachenmeier 2004; Lachenmeier et al. 2003, 2005, 2006, 2007; Bauer-Christoph and Lachenmeier 2005)]. Both devices were equipped with an alcohol extension set, which consists of a blind cap for the NaOH inlet (which is unnecessary for alcohol distillation and may lead to losses) and an outlet tube with reduced diameter to facilitate the use of a measuring flask as receiver. Both instruments were obtained from C. Gerhardt GmbH & Co. KG, Königswinter, Germany. The steam generator water supplies were coupled to tanks filled with distilled water. The coolant inlets and outlets were attached to the laboratory’s central coolant water system temperated at 10 °C. Before every start-up, the steam generators were pre-heated with a water sample at full steam power (according to the manufacturers’ instruction). For the tempering of the sample the heating circulator bath Haake DC10-W26 (Thermo Scientific, Braunschweig, Germany) was used. The determination of density was accomplished with the density meter DE51 with SC30 sample changer by Mettler-Toledo (Giessen, Germany). The instrument was adjusted with air and water according to the manufacturer. The adjustment was checked daily using water standards. The sample temperature for all measurements was adjusted to 20 °C. The alcoholic strength was calculated automatically from the measured density using the stored official alcohol table data. Between measurements, the connecting tubes were purged with air. Before shutdown of the system, the hoses were cleaned with distilled water and purged with acetone and air until they were dry.

### Sample preparation and measurement

The sample preparation was conducted as previously described (Lachenmeier et al. 2006). In short, the sample was temperated in a water bath at 20 °C. Following this, the sample was pipetted into a 250 mL Kjeldatherm digestion tube. Rests of the sample sticking to the edge of the tube were rinsed down with distilled water. Subsequently the tube was clamped in the distillation device. After placing a graduated flask filled with 3 mL of distilled water under the distillate outlet tubing, the program was started, and the distillation was automatically performed while occasionally shaking the receiver flask. After termination, the receiver and the tube were replaced and the hoses were rinsed with distilled water to be ready for the next sample. The graduated flask with the distillate was shaken, temperated in a water bath at 20 °C and filled up to the calibration mark with water (20 °C) and again shaken. The shaking steps of the flask are absolutely essential because a considerably inhomogeneity of the solution has been observed following distillation, which may lead to errors up to 5 % vol if the solution is not carefully homogenized. An aliquot of the distillate (about 20 mL) was filled into glass vials placed into the autosampler and the alcoholic strength was automatically determined with the oscillation-type density meter. As alternative to pipetting, samples (temperated at 20 °C) may be filled in graduated flasks, followed by filling the content of the flask into the Kjeldatherm digestion tube. The residues in the flask then need to be carefully rinsed 3 times with distilled water into the Kjeldatherm digestion tube.

### Optimisation of the distillation procedure

The influence of all basic operation parameters of the method including the steam power of the distillation device, the time of distillation, sample volume, receiver volume, sample preparation (pipetting or measuring flask), and cooling water flow through the cooling coil were examined. As we have previously shown that some of these settings show interactions, and a partly non-linear behaviour (Lachenmeier et al. 2006), experimental designs using D-optimal or central composite designs were used (Montgomery 2005). The designs and calculations were conducted using the Design Expert V7.0.0 software (Stat-Ease Inc., Minneapolis, MN, USA).

The following experiments were conducted:

1. Optimal working settings for steam power, sample volume and distillation time were initially investigated. The same central composite design as described previously (Lachenmeier et al. 2006) was used for this purpose. The steam power was varied at levels of 40, 45, 65, 85 and 100 %, the distillation time was varied at levels of 20, 44, 80, 116 and 140 s, and the sample pipetting volume was varied at 5, 14, 28, 41 and 50 mL. The experiment (n = 20) was conducted at both instruments using the same fruit liqueur.
2. The new instrument allows to adjust the setting of coolant flow at 2, 5 and 7 L/min. The standard setting is 5 L/min. To research the influence of coolant flow setting, and additionally again the steam power, a central composite design was used. The cooling flow setting was varied at the two levels 5 and 7 L/min, while the steam power was varied at levels of 70, 75, 85, 95 and 100 %. The experiment was conducted only with the new instrument using a cream liqueur (n = 15).
3. To research the influence of sample preparation, a d-optimal response surface design was used. The sample volume was varied at levels of 10, 25, 50, 100 and 200 mL. The receiver volume (volume of graduated flask) was set at either exactly the same volume as the sample volume, or at the double volume (for example, 25 mL of sample were either distilled into 25 or 50 mL). Finally as third factor, volumetric glass pipettes or graduated flasks were used to transfer the sample volume into the Kjeldatherm tube.

### Validation and statistics

As first step of the validation, the requirement of the EU reference method was verified (European Commission 2000). For this, non-denatured food-grade neutral alcohol (96 % vol) was diluted to 50 % vol and measured before and after distillation using density measurement. The absolute loss of the distillation step was then calculated to compare with the requirement of a loss of alcohol of not more than 0.1 % vol.

Linearity in the working range of liqueur analysis was assessed by diluting non-denatured food-grade neutral alcohol (96 % vol) with distilled water to concentrations between 0.5 and 35 % vol. The alcoholic strength of the dilutions was determined before and after distillation using density measurement. Linear regression analysis was used to compare both measurement series. The root mean squared error (RMSE) was calculated to estimate bias.

To determine the performance of the method, precision as expressed by the relative standard deviation (RSD = standard deviation (SD)/mean × 100) of analyzing authentic samples was determined under repeatability conditions (analysis within short time intervals), and under within-laboratory reproducibility conditions (analysis on different days). As further validation parameters, the repeatability (r) and reproducibility (R) were calculated as SD × 2.8.

All calculations were conducted with Origin Pro v7.5 software (OriginLab Corporation, Northampton, MA, USA). Statistical significance was assumed at below the 0.05 probability level.

## Results

The basic operating parameters of the steam distillation device were assessed using three experimental designs. By means of response surface analysis, the regression coefficients of the models are determined and the statistical analysis of variance (ANOVA) approach calculates the individual significance of each coefficient. Table 1 lists the regression coefficients of all experiments.

**Table 1:** Regression coefficients in coded values for the optimisation of steam distillation on different instruments

Regression coefficient	Experiment 1 A-Steam power B-Distillation time C-Sample volume		Experiment 2 A-Steam power B-Coolant flow	Experiment 3 A-Sample volume B-Receiver volume C-Pipette/flask
Device	Vapodest 30	Vapodest 200	Vapodest 200	Vapodest 200
Matrix	Liquorice liqueur		Cream liqueur	Cream liqueur
Intercept	16.89	19.96	16.77	16.63
A	1.92**	1.39	0.03	0.43**
B	3.26***	4.41***	0.02*	−0.25*
C	−0.20	−0.70	–	−0.35**
A^2^	−0.82	−0.33	−0.04	−0.084
B^2^	−2.34***	−2.87***	no effect	–
C^2^	−0.21	−0.09	–	–
AB	−1.57*	−1.45	−0.02	0.30*
AC	−0.85	−1.10	–	0.31*
BC	0.36	−0.18	–	0.14
r^2^	0.947**	0.949***	0.605*	0.842**

* P ≤ 0.05, ** P ≤ 0.01, *** P ≤ 0.001

The first experiment investigated steam power, sample volume and distillation time. The factors are given in coded values, which make the models directly comparable between each other and offer the opportunity to find the importance of each regression term in the model. Significant differences between the two steam distillation devices were found. The old instrument (Vapodest 30) showed a significant influence of steam power and distillation time, an additional quadratic influence of distillation time, as well as an interaction between steam power and distillation time. The new instrument (Vapodest 200) only showed a significant influence of distillation time as well as an additional quadratic influence of distillation time.

In the second experiment, the influence of the new possibility to regulate the coolant flow was investigated (which was not possible with the old instrument). The results (Table 1) show that the coolant flow indeed has a slight but significant influence. The higher setting (7 L/min) improved the alcohol recovery. The second experiment also confirmed the lack of influence of the steam power for the new instrument.

The third experiment (Table 1) showed that the sample preparation has small but significant influence on the results. The highest influence was found for the sample volume. Using only 10 mL of sample was found to result in more imprecise measurements. Small differences were found for receiver volume (double volume is better) and the use of pipettes or flasks for sample transfer (pipette use is better). Some interactions were found between sample volume and receiver volume (at lower volumes, the use of smaller receiver volumes is overproportionally worse) and between sample volume and use of pipettes or flasks (at lower volumes, the use of flasks is overproprotionally worse than the use of pipettes).

The method validation was conducted using the following parameters based on the optimization experiments: steam power at 70 %, sample volume 25 mL transferred using pipettes, receiver volume 50 mL. The absolute loss of alcohol for a solution at 50 % vol was 0.10 % vol. The determination was linear over the whole working range (R^2^ = 0.9999, p < 0.0001) with a RMSE of 0.03 % vol.

The optimised procedure was validated with 4 different liqueurs covering the typical range of these products between 15 and 35 % vol (Table 2). For all analysed products, the method shows an adequate precision, with relative standard deviations below 0.4 % (intraday) and below 0.6 % (interday). The absolute standard deviations were between 0.06 and 0.08 % vol (intraday) and between 0.07 and 0.10 % vol (interday).

**Table 2:** Validation results for alcoholic strength measured with steam distillation and oscillation-type densimetry

Sample	Repeatability conditions (n = 10)				Within-laboratory reproducibility conditions (n = 30)			
	Mean (% vol)	SD (% vol)	r (% vol)	RSD [%]	Mean (% vol)	SD (% vol)	R (% vol)	RSD [%]
Cinnamon whisky liqueur	33.46	0.06	0.17	0.18	33.44	0.10	0.28	0.30
Black currant liqueur	15.18	0.05	0.14	0.33	15.15	0.07	0.20	0.46
Liquorice liqueur	20.20	0.08	0.22	0.40	20.15	0.09	0.25	0.45
Whisky cream liqueur	17.19	0.06	0.17	0.35	17.17	0.10	0.28	0.58

## Discussion

A major difference between the old and the new device was the improvement of the power of the steam generation (old device: 1600 W, new device: 2200 W), which led to a robust and complete alcohol distillation that now is almost independent of the steam power setting. These results confirm our previous finding using a steam distillation device of another manufacturer (2200 W), which showed a similar improvement in distillation behaviour (Lachenmeier et al. 2006). The setting of 70 % steam power for our further experiments was chosen to avoid a very rapid distillation at the beginning of the distillation, which could potentially exceed the capacity of the condenser leading to a warmer distillate and potential losses (especially for high-strength beverages). Using the old device, we had always recommended to fill an amount of water in the receiver to avoid evaporation at the beginning of the distillation, when the liquid was comparably warm with high concentrations of ethanol. With the new device, we have upheld this protocol to fill water into the receiver, so that the distillation outlet tube is immersed in the water. Experiments (data not shown) have found that the water in the receiver is not absolutely necessary due to the improved condenser, but we have decided to still follow the old protocol to be on the safest possible side and to allow the switching between devices without changes in the standard operating procedure that may lead to operator errors.

The significant influence of distillation time (including a quadratic influence) also corroborates our previous findings (Lachenmeier et al. 2006). It is clearly deleterious to remove the receiver too early during the distillation, when the ethanol transfer is not complete. No changes in alcohol content were observable at distillation times of 80 s or longer, but we typically recommend to distil as long as just below the calibration mark of the receiver to ensure a complete distillation, e.g. also in cases of spirits with higher alcoholic strengths (such as absinthes). For the same reason, it appears to be advantageous to shake the receiver during the distillation process, which may reduce the vapour pressure of ethanol due to immediate dilution, especially at the start of the distillation, and also reduce the volume due to the volume contraction effect of ethanol–water mixtures.

The influence of sample volume may derive from a larger volume error at smaller volumes, rather than problems in the distillation itself. It is suggested to use volumes of more than 10 mL. This confirms our previous results on egg liqueur, which showed inacceptable precision if only 5 or 10 g of sample were used (Lachenmeier et al. 2005). While we found no significant differences for a volume of 200 mL, we believe that such high volumes should not be used, because especially for spirits and liqueurs with higher alcoholic strengths, the distillation time may not be sufficient to recover the complete amount of alcohol. For the same reason, we suggest to use a larger receiver volume than the original sample volume. The error increase due to dilution appears to be compensated by the more complete distillation.

Due to the instrumental improvements, the method has become more rugged than before, and there are only few influences that would lead to incomplete distillation. The major parameters are monitored by the software during operation and in case of deviations (e.g. lack of steam power) warnings are displayed. Our validation parameters have shown that the performance of the method corresponds to the data presented for the reference method and we believe that automated steam distillation, while not strictly mentioned in the reference procedure, can be used for the purpose.

The excellent reproducibility of the procedure can be explained by the full automation of the distillation as well as the density measurement. A manual density measurement (either by pycnometry or by manual oscillation-type densimetry) was previously found as leading to higher errors (Lachenmeier et al. 2005).

The validation data with repeatability limits (r) in the range of 0.14–0.22 % vol compare well to the repeatability limits found in the literature. For example, the German reference procedure [distillation/pycnometry (Anon. 1982)] reported r = 0.19 % vol, while the EU reference method (European Commission 2000) reported r = 0.30 % vol (distillation/pycnometry) and r = 0.12 % vol (distillation/electronic densimetry). Our previous validation data using steam distillation (old instrument) were also very similar [r range 0.15–0.23 % vol (Lachenmeier et al. 2003)]. Because the method’s precision was already very high using the old instrument, and further improvement was not detectable using the new instrument, it is suggested that the major part of the method uncertainty derives from factors apart from the distillation step such as the uncertainty of the density measurement as well as the volumetric errors in pipetting and filling up the graduated flaks with the distillate, which appear to have the highest influence. Indirect evidence for this finding is also provided by the fact that infrared spectroscopic methods for quantifying alcoholic strength (which are conducted in the beverage itself without any need for sample preparation) usually had a much lower repeatability [for example, r = 0.02 % vol for both spirits (Lachenmeier 2007) and wine (Patz et al. 2004), and 0.05 % vol for liqueurs (Arzberger and Lachenmeier 2008)].

## Conclusions

Improved automatic steam distillation devices offer an excellent alternative for sample cleanup of volatiles from complex matrices. The major advantages are the low costs for consumables per analysis (only distilled water is needed) compared to techniques such as liquid–liquid extraction (which requires solvents) or solid-phase extraction (which requires extraction tubes). Besides for alcohol distillation, we expect that the new devices may also improve other challenging steam distillation applications such as the determination of formaldehyde (Jendral et al. 2011) or dithiocarbamates (Rai et al. 2012).
